# King Edward's Hospital Fund for London

**Published:** 1904-12-24

**Authors:** 


### HOSPITAL ADMINISTRATION.

### CONSTRUCTION AND ECONOMICS.

### KING EDWARD'S HOSPITAL FUND FOR LONDON.

DISTRIBUTION MEETING.

A meeting of the General Council of King Edward's Hospital Fund for London for the purpose of awarding grants to the hospitals and convalescent homes for the present year was held on December 19th at Marlborough House, the Prince of Wales being in the chair.

Those present were:—The Bishop of London, the Bishop of Rochester, the Chief Rabbi (the Rev. Dr. Adler), Lord Rothschild, Lord Iveagh, the President of the Hospital Sunday and Hospital Saturday Funds (the Lord Mayor), the President of the Royal College of Physicians (Sir W. S. Church, Bart.), the President of the Royal College of Surgeons (Mr. John Tweedy), the Right Hon. Sir Savile Crossley, Bart., M.P., the Right Hon. Sir Joseph Dimsdale, Bart., M.P., the Right Hon. C. Stuart Wortley, K.C., M.P., Sir Trevor Lawrence, Bart., Sir John Aird, Bart., M.P., Sir Henry Burdett, K.C.B., Sir W. J. Collins, Sir John Craggs, Mr. F. M. Fry, Mr. J. Pierpont Morgan, jun., Mr. J. Danvers Power, Mr. Albert G. Sandeman, Mr. H. C. Smith, Mr. Edgar Speyer.

The minutes of the last meeting having been read and confirmed, letters of regret were reported from the following:—The Lord-Lieutenant of London (the Duke of Fife), the Lord-Lieutenant of Middlesex (the Duke of Bedford), Archbishop Bourne, the Rev. T. Bowman Stephenson, D.D., Viscount Duncannon, Lord Strathcona and Mount Royal, Lord Farquhar, the Postmaster-General, the Chairman of the County Council, the Governor of the Bank of England, Mr. Thomas Burt, M.P., Mr. Sydney Buxton, M.P., Mr. William Latham, K.C., Mr. Julius Wernher.

#### The Treasurer's Statements.

The Honorary Treasurer (Lord Rothschild) stated that the funds received for general purposes up to December 13, after paying expenses, were £67,999 18s. 11d., and that the League of Mercy would provide £14,000 this year. Reviewing the figures, Lord Rothschild said that his Royal Highness would remember that on the previous occasion the Council thought right to trench on their reserves so as to increase the contributions to the hospitals to £100,000. On this occasion the Council proposed only to distribute the money they had in hand, and not to trespass on reserve. He regretted that there was this year very little difference in the regular contributions to the Fund, but the League of Mercy had been very active, and had been able to contribute a larger sum to the Fund than they did last year.

Sir Henry Burdett said:—It would be remembered by his Royal Highness and the Council that when his Majesty the King issued his first appeal with regard to the Fund, he expressed a wish that the income should in part be made up of annual subscriptions of small sums. The League of Mercy endeavoured to carry out this object, as would be seen from the report of the League, which was working on those lines, for it received one shilling subscriptions in contributions of one penny a month. As one example it might be mentioned that 32,000 pence were collected in one tenement occupied by artisans in sums of under one shilling, and collected by one penny subscriptions, and few, if any, could give more. This was an example of how the League benefited by small contributions. The League was fulfilling in a wonderful way the wishes of his Majesty the King. The percentage of the increase comparing 1903 with 1902 was 54 per cent., while the percentage of increase between 1904 and 1902 was 84 per cent. He was glad to report that the total result of the collections for this year would be £16,000, and it had been decided that the League should hand to the treasurer £14,000 as a result of the work of the League of Mercy. With his Royal Highness's sanction he might mention that the League of Mercy, which extended to the Home Counties, had very usefully and helpfully inspected and given grants to those hospitals outside the area of the county of London to which the King's Fund did not contribute. He paid a warm tribute to Dora Countess of Chesterfield, the president of the South Kensington branch, which had this year collected upwards of £1,280, showing that it was possible to raise in small subscriptions in a division more than £1,200, the maximum originally fixed for each district; to H.R.H. the Princess Victoria of Schleswig-Holstein, who, as president of Berkshire, had increased the contributions there from £16 in 1903 to £777 in 1904; and to H.R.H. the Duchess of Teck for the great success achieved in the Epsom-Esher division of Surrey, of which she was president. The League united all classes, from the highest to the lowest. All the workers connected with it were entitled to the warmest praise, and he was confident the League had now been established upon a permanent basis.

He might take this opportunity of stating that no one but the Executive Committee of the League of Mercy and those associated with that body could adequately realise how much good the influence and generosity of their Royal Highnesses the Prince and Princess of Wales had helped the movement.

#### Report of the Executive Committee.

The Chairman of the Executive Committee (Mr. H. C. Smith) presented the report of the Executive Committee as follows:—

"May it please your Royal Highness,—

"The Executive Committee recommend this year the distribution of £79,000 to the London Hospitals, making with the £1,000 entrusted to the Fund by the London Parochial Charities for the Convalescent Homes a total distribution of £80,000.

"The Committee feel that, much as they would have liked to recommend a distribution of £100,000 again this year, they would not be justified in contemplating a further depletion of the capital of the Fund the interest of which forms so considerable a part of the annual income.

"The Committee regret that they have been unable to raise the sum (equal to at least £300,000) which is necessary in order to enable your Royal Highness to take advantage of the munificent offer of an anonymous donor. In a year during which so many other pressing appeals have been before the public the task was beyond their powers, and recognising this they thought it better not to enter upon the extensive operations which are in any case inseparable from the collection of a sum of such magnitude from the general public. They have, however, lost no opportunity of bringing the matter to the notice of persons with whom the ordinary business of the Fund brings them into contact, and in addition three appeals have been made by means of letters to the various newspapers.

"The reference in the anonymous donor's letter to the possible diversion of funds subscribed for the relief of the sick poor to purposes of medical education has not been lost sight of. A committee, consisting of Lord Welby, Sir Edward Fry, and the Bishop of Stepney, have been appointed to inquire into the subject, and their report may be expected to be published before the end of the year. The Executive Committee are glad to have been able to secure the services of men so experienced in public business."

Report of the Distribution Committee.

The Chairman of this Committee (Sir William Church) presented the Report as follows:—

"The Committee have the pleasure to report that the Executive Committee, with the approval of his Royal Highness the President, placed in their hands this year the sum of £79,000 for distribution among the London hospitals.

"In connection with the grants which the Committee are able to recommend for the year 1904 it may be well to mention that the amount available for distribution in 1903 was £20,000 more than that which is now at disposal, viz. £99,000, of which a sum of £18,751 9s. was transferred from the general funds.

"It will be remembered that in 1903 the Committee set aside £10,500 to be drawn on as required for the expenses incidental to the amalgamation of certain hospitals. They noted with satisfaction in their last Report that the National Orthopædic and the Royal Orthopædic Hospitals had amalgamated, and expressed their regret that the City Orthopædic Hospital had not consented to join in the amalgamation. They now learn that the City Orthopædic Hospital continues to stand out of this most desirable amalgamation, and but for the fact that the annual grant of £250 made to this hospital was for the maintenance of beds reopened by the Fund they would have seriously considered the question of making any grant. They have, however, felt bound this year, while withdrawing the annual grant, to make a donation of £250, and must reserve their entire freedom of action next year in considering the claims of the City Orthopædic Hospital on the Fund.

"The Committee draw attention to the undesirability of opening new special hospitals with small accommodation, and they will feel it their duty to discourage further attempts in this direction.

"Several of the subjects which, being preliminary to modern medical education, are taught at the various medical schools, occasion a demand on the accommodation of the hospitals and a drain on the resources of the schools which would be avoided were instruction on these subjects provided elsewhere. The question is one which deserves the attention of all who are concerned with the increasing requirements of hospitals.

"The number of beds reopened as free beds by the Fund since its inauguration is 443.

"The Committee have again done their utmost to show appreciation of the scheme for the removal of King's College Hospital.

"An application was received from St. Bartholomew's Hospital for a grant towards the Rebuilding Fund, accompanied by an intimation to the effect that when the necessary money to reconstruct the hospital had been collected no further appeal would be made for public assistance. The Committee having no precedent to guide them, and being in doubt as to their powers to make a grant in these circumstances, referred the matter to the Executive Committee. That Committee resolved as follows:—

"'That, in the opinion of the Executive Committee, the King's Fund cannot properly be devoted to capital expenditure on behalf of hospitals possessed of endowments substantially adequate to their maintenance, as such a departure would preclude the regular practice of the Fund in associating annual inspection with contribution.'"

"The Distribution Committee therefore regret that in accordance with this instruction, they have no power to consider the application for a grant made by this ancient foundation, the work of which has for so long a period been of the greatest value to the sick poor of London. They desire to add that the hospital was inspected by the Fund and that the report received from the visitors was favourable.

"Among the 106 reports of the visitors which have, as usual, been of much service to the Committee, they have observed in several cases that recommendations have been made in connection with the existing arrangements for protection against fire. This subject appears to them to be one which might usefully occupy the attention of the Executive Commitiee, or of a special committee.

"In addition to their published remarks, the Committee have followed their usual practice in recommending that various suggestions should be made privately to many of the hospitals, especially in connection with expenditure which in some cases still appears to be excessive.

"The details of the awards to the various hospitals are appended."

---

LIST OF GRANTS TO THE HOSPITALS.

Sir Savile Crossley read the awards as follows:—

The Distribution Committee desire to draw attention to the fact that the absence of a grant does not necessarily imply dissatisfaction.

Alexandra Hospital for Children, Queen Square, Bloomsbury, W.C.—£500 donation; £350 for balcony.

Belgrave Hospital, Clapham Road, Kennington. — £500 donation.

Blackheath and Charlton Cottage Hospital, Shooter's Hill Road, Blackheath, S.E.—£25 donation.

Bolingbroke Hospital, Wandsworth Common, S.W.—£2,000 donation to new Building Fund.

British Lying-in Hospital, Endell Street, St. Giles, W.C.—£100 donation.

Cancer Hospital, Fulham Road, Brompton, S.W. — An excellent institution.

Central London Ophthalmic Hospital, 238A Gray's Inn Road, W.C.—£150 donation.

Central London Throat and Ear Hospital, Gray's Inn Road, W.C.—£300 donation; the Committee would view with satisfaction an amalgamation of the Throat and Ear Hospitals.

Charing Cross Hospital, Agar Street, West Strand, W.C.—£2,500 donation; £1,000 to building.

Chelsea Hospital for Women, Fulham Road, S.W.—£350 donation.

Cheyne Hospital, Cheyne Walk, Chelsea, S.W. — £100 donation; in consideration of the fact that curable cases are admitted.

City of London Hospital for Diseases of the Chest, Victoria Park, E. — £1,500 annual; £750 donation. £1,500 annual to support beds reopened by this Fund.

City of London Lying-in Hospital, City Road, E.C.—An institution doing excellent work.

City Orthopædic Hospital, 26 and 27 Hatton Garden, E.C.—£250 donation; the Committee feel bound to make this donation in order to provide for the cost of beds opened by this Fund up to December 31st. In view of the Hospital's refusal to join in the amalgamation of the Orthopædic Hospitals the Committee reserve entire freedom of action in respect of next year's awards.

Clapham Maternity Hospital, 41 and 43 Jeffreys Road, Clapham, S.W.—£100 donation.

"Dreadnought" Hospital, Greenwich, S.E.—£500 annual; £1,000 donation.

East End Mothers' Home, 395 and 396 Commercial Road, E.—£200 donation.

East London Hospital for Children, Glamis Road, Shadwell, E.—£1,000 donation.

Eltham and Mottingham Cottage Hospital, High Street, Eltham.—£25 donation. An institution deserving of support.

Evelina Hospital, Southwark Bridge Road, S.E.—£100 donation.

Free Home for the Dying, 29 North Side, Clapham Common, S.W.—The Committee are pleased to see that this useful institution is well supported.

French Hospital, 172 Shaftesbury Avenue, W.C.—£100 donation.

Friedenheim Hospital, Upper Avenue Road, Swiss Cottage, N.W.—£25 donation.

General Lying-in Hospital, York Road, Lambeth, S.E.—£200 donation.

German Hospital, Dalston Lane, N.E.—£150 donation. Towards new mortuary and post-mortem room.

Gordon Fistula Hospital, Vauxhall Bridge Road, S.W.—£50 donation.

Great Northern Central Hospital, Holloway Road, N.—£750 annual, to maintain beds reopened by this Fund; £1,000 donation (£250 for improvements now in hand).

Grosvenor Hospital for Women and Children, Vincent Square, Westminster, S.W.—£100 donation.

Guy's Hospital, St. Thomas Street, Borough, S.E.—£5,000 annual; £1,000 donation.

Hampstead General Hospital, Parliament Hill, N.W.—£500 donation to Building Fund.

Home and Infirmary for Sick Children, Sydenham Road, Lower Sydenham, S.E.—£25 donation towards cost of balcony.

Hospital for Consumption, Brompton, S.W.—£350 donation towards ward furniture.

Hospital for Diseases of the Throat, Golden Square, W.—£100 donation. The Committee would view with satisfaction an amalgamation of the Throat and Ear Hospitals.

Hospital for Epilepsy and Paralysis, 4 Maida Vale, W.—£400 donation.

Hospital for Invalid Gentlewomen, 90 Harley Street, W.—£200 donation.

Hospital for Sick Children, Great Ormond Street, Bloomsbury, W.C.—£500 annual to maintain beds opened by this Fund; £500 donation.

Hospital for Women, Soho Square, W.—£1,100 donation to maintain beds reopened by this Fund. The Committee draw attention to the desirability of removing this hospital to another site.

Hospital of St. John and St. Elizabeth, Grove End Road, St. John's Wood.—£500 annual to maintain beds reopened by this Fund.

Italian Hospital, Queen Square, Bloomsbury, W.C.—£500 annual to maintain beds reopened by this Fund.

Kensington Dispensary and Children's Hospital, 49 Church Street, Kensington, W.—£25 donation.

King's College Hospital, Portugal Street, Lincoln's Inn, W.C.—£1,000 annual; £3,500 donation—£2,500 to the Fund for removal of the hospital to South London, which the Committee view with great satisfaction.

London Fever Hospital Liverpool Road, Islington, N.—£200 donation.

London Homœopathic Hospital, Great Ormond Street, W.C.—£200 donation.

London Hospital, Whitechapel Road, E.—£5,000 annual to meet expenditure out of capital made necessary by improvements in the wards; £2,000 donation.

London Lock Hospital (Female), Harrow Road, W.—£600 donation.

London Temperance Hospital, Hampstead Road, N.W.—£400 donation.

London Throat Hospital, 204 Great Portland Street, W.—£50 donation. The Committee would view with satisfaction an amalgamation of the Throat and Ear Hospitals.

Medical Mission Hospital (formerly entered as Cottage Hospital, Canning Town), 538-540 Barking Road, E.—£100 donation.

Memorial Cottage Hospital, Mildmay Park, N.—£25 donation.

Metropolitan Ear, Nose, and Throat Hospital, 64 Grafton Street, Fitzroy Square, W.—The Committee are unable to make a grant to this institution as a hospital, but would be prepared to consider a grant in the event of amalgamation with the other Throat and Ear Hospitals.

Metropolitan Hospital, Kingsland Road, N.E.—£500 annual to maintain beds reopened by this Fund; £1,500 donation.

Middlesex Hospital, Mortimer Street, W.—£1,000 annual to meet deficiency; £1,250 donation.

Mildmay Mission Hospital, Austin Street, Bethnal Green, E.—£200 donation.

Miller Hospital, Greenwich Road, S.E.—£750 donation.

Mount Vernon Hospital for Consumption (formerly entered as North London Hospital for Consumption), Mount Vernon, Hampstead, N.W.—£1,000 annual to support beds reopened by this Fund; £500 donation.

National Dental Hospital, 187-191 Great Portland Street, W.—£25 donation to debt.

National Hospital for Diseases of the Heart, 32 Soho Square, W.—£15 donation to complete cost of $x$-ray apparatus.

National Hospital for the Paralysed and Epileptic, Queen Square, Bloomsbury, W.C.—£750 annual to meet deficiency; £1,000 donation.

National Orthopædic Hospital, 234 Great Portland Street, W.—£400 donation. This grant is given pending the amalgamation with the Royal Orthopædic Hospital which the committee view with great satisfaction and for which a special sum is set aside.

New Hospital for Women, 144 Euston Road, N.W.—£250 donation.

North-Eastern Hospital for Children, Hackney Road, Bethnal Green, E.—£500 annual; £1,000 donation.

North-West London Hospital, 18-22 Kentish Town Road, N.W.—£500 donation. The committee again urge that immediate steps be taken to provide funds not only to maintain but to rebuild this hospital.

Norwood Cottage Hospital, Hermitage Road, Central Hill, Upper Norwood, S.E.—£25 donation.

Oxygen Hospital, 2 Fitzroy Square, W.

Paddington Green Children's Hospital, Paddington Green, W.—£400 donation.

Passmore Edwards Acton Cottage Hospital, Gunnersbury Lane, Acton, W.—The Committee are glad to say that it does not require help from the Fund this year.

Passmore Edwards Hospital, Shrewsbury Road, East Ham.—£100 donation (£50 to out-patient department improvements).

Passmore Edwards Hospital for Willesden, Harlesden Road, N.W.—£50 donation.

Passmore Edwards Cottage Hospital, Bounds Green Road, Wood Green, N.—£25 donation.

Poplar Hospital, Blackwall, E.—£500 annual.

Queen Charlotte's Lying-in Hospital, Marylebone Road, N.W.—£500 donation.

Queen's Jubilee Hospital, Richmond Road, Earl's Court, S.W.—£200 donation to building fund.

Royal Dental Hospital of London (formerly entered as Dental Hospital), Leicester Square, W.C.—£250 donation to reduce debt.

Royal Ear Hospital, Frith Street, Soho Square, W.—£500 donation to building. The Committee would view with satisfaction an amalgamation of the Throat and Ear Hospitals.

Royal Eye Hospital, St. George's Circus, Southwark, S.E.—£250 donation.

Royal Free Hospital, Gray's Inn Road, W.C.—£750 annual to meet deficiency; £1,500 donation towards improvements. The Committee are glad that the improvements previously referred to have been carried out.

Royal Hospital for Diseases of the Chest, 231 City Road, E C.—£400 donation.

Royal London Ophthalmic Hospital, City Road, E.C.—£2,000 to help to maintain the beds opened by this Fund; £2,000 donation. The Committee consider this hospital should receive more support from the public.

Royal Orthopædic Hospital, 297 Oxford Street, W.—£200 donation. This grant is given pending the amalgamation with the National Orthopædic Hospital, which the Committee view with great satisfaction, and for which a special sum is set aside.

Royal Waterloo Hospital for Children and Women, Waterloo Bridge Road, S E.—£750 donation for building.

Royal Westminster Ophthalmic Hospital, King William Street, West Strand, W.C.—£300 donation to debt.

St. Barnabas Hospital, 9 Lloyd Street, W.C.—This institution does not appear to come within the scope of the King's Fund, not being managed by a duly appointed Committee.

St. Bartholomew's Hospital, West Smithfield, E.C.

St. George's Hospital, Hyde Park Corner, S.W.—£1,000 donation.

St. John's Hospital for Diseases of the Skin, out-patient department 49 Leicester Square, W.C., in-patient department 238 Uxbridge Road, W.—£25 donation to Shepherd's Bush branch.

St. John's Hospital, Morden Hill, Lewisham, S.E.—£75 donation towards renovating nurses' quarters.

St. Mark's Hospital, City Road, E.C.—£75 donation.

St. Mary's Children's Hospital, Plaistow, E.—£600 donation.

St. Mary's Hospital, Praed Street, Paddington, W.; £1,000 annual to meet deficiency; £2,000 donation (£1,000 to be applied towards completion of buildings).

St. Monica's Hospital, Brondesbury Park, N.W.—£60 donation.

St. Peter's Hospital for Stone, Henrietta Street, Covent Garden, W.C.—£75 donation.

St. Saviour's Hospital, Osnaburgh Street, Regent's Park, N.W.—£100 donation.

St. Thomas's Hospital, Albert Embankment, S.E.—The Committee are glad to see that there is a substantial surplus this year.

Salvation Army Maternity Hospital, Ivy House, 271 Mare Street, Hackney, N.E.—The Committee cannot continue to support this institution in its present condition.

Samaritan Free Hospital, Marylebone Road, N.W.; £500 annual.

Santa Claus Home, Cholmeley Park, Highgate, N.—£25 donation, in consideration of the fact that curable cases are admitted.

South Wimbledon and Merton Cottage Hospital, 173 Merton Road, Wimbledon.

Tottenham Hospital, Tottenham, N.—£1,000 annual to maintain beds reopened by this Fund; £2,000 donation (£1,000 to building fund).

University College Hospital, Gower Street, W.C.; £1,000 annual.—£2,000 donation. The Committee trust that this Hospital will now be able to reopen the remaining 19 closed beds.

Victoria Hospital for Sick Children, Tite Street, Chelsea, S.W.—£1,400 donation (£1,000 to building fund) The Committee trust that the vacant wards will shortly be opened for free patients.

West End Hospital for Diseases of the Nervous System, 73 Welbeck Street, W.—£125 donation towards balcony.

Western Ophthalmic Hospital, 153 and 155 Marylebone Road, W.—£150 donation to out-patients' department.

West Ham Hospital, Stratford, E.—£300 annual towards maintenance; £2,000 donation (£1,000 to building).

West London Hospital, Hammersmith Road, W.—£1,500 annual; £500 donation.

Westminster Hospital, Broad Sanctuary, S.W.—£750 annual; £750 donation.

Woolwich and Plumstead Cottage Hospital, Shooter's Hill, Kent.—£50 donation.

Total — Annual, £28,300; donations, £50,700. Grand total, £79,000.

### Grants to Convalescent Institutions.

In the absence of Mr. Wm. Latham, K C. (Chairman of the Convalescent Homes Committee), Mr. F. M. Fry read the Report as follows:—

Owing to the continued liberality of the Trustees of the London Parochial Charities the Committee have again £1,000 to distribute.

The Committee have followed the course adopted in previous years of making out of the limited sum at their disposal fairly substantial grants to a comparatively small number of institutions, and to a great extent they have repeated grants to those before approved of in preference to varying the distribution.

The Committee desire to call attention to the expediency of keeping separate accounts of Convalescent Homes.

The following awards were recommended:—

All Saints Convalescent Hospital, Eastbourne.—£100.

Alexandra Hospital, Convalescent Home at Painswick.—£50.

Charing Cross Hospital, Convalescent Home at Limpsfield, Surrey.—£50.

East London Hospital for Children, Convalescent Home at Bognor.—£100.

King's College Hospital, Convalescent Home at Hemel Hempstead.—£100.

Mary Wardell Convalescent Home, Stanmore, Middlesex.—£50.

Metropolitan Hospital, Convalescent Home at Cranbrook.—£25.

Metropolitan Convalescent Institution, 32 Sackville Street, Piccadilly, W.—£200.

Middlesex Hospital, Convalescent Home at Clacton-on-Sea.—£100.

Mrs. Gladstone's Free Convalescent Home for the Poor, Ravensbury House, Mitcham, Surrey.—£25.

Mrs. Kitto's Free Convalescent Home, South Park, Reigate.—£25.

National Hospital for the Paralysed and Epileptic, Convalescent Home at East Finchley.—£50.
Paddington Green Children's Hospital, Convalescent Home at Slough.—£50.
Queen Charlotte's Lying-in Hospital, Convalescent Home at 20 Victoria Road, Kilburn, N.W.—£25.
Victoria Hospital for Children, Convalescent Home at Broadstairs.—£50.
Total grants to Convalescent Homes, £1,000.

### SPEECH BY THE PRINCE OF WALES.

It is my pleasing duty, as President of the Fund, to move the adoption of the Report of the Distribution Committee, with that of the Executive Committee, which we have just heard read.

### The League of Mercy.

In so doing I note with pleasure that, as we are receiving a larger sum from the League of Mercy than we anticipated, it is not now necessary to draw on our reserve, as we at first feared, in order to distribute £80,000, and, in fact, I am glad to say we have a small surplus, which will be used towards replacing, though only to a slight extent, the £18,000 odd taken from the reserve last year.

Let me take this opportunity of expressing the deep gratification we all feel that the League of Mercy is growing in strength and popularity, and I should like to congratulate those whose labours have produced this result. It is satisfactory to feel that the League is becoming firmly established, and its work more understood and appreciated.

### Results of the Year 1904.

In reviewing the results of this year's work I must refer to what was said last year, when we distributed £100,000 by drawing on our available balance for that purpose to the extent of £18,751 9s. I then pointed out that we drew that large sum from our reserves for one year only, and I expressed a hope that we might receive increased contributions to enable us to distribute an equal sum this year, without any encroachment on our reserves. These hopes were subsequently further raised by the munificent offer of an anonymous donor, who promised us a gift which would increase our permanent income by £4 668 a year, provided twice this amount were raised before the end of this year. That condition unfortunately has not been fulfilled. As I anticipated last year, the public are now confronted with appeals for over £1,000,000 in connection with hospitals in London. There is, of course, a limit to what the charitable people can do, and we have had to cut our coat according to our cloth. We are only able this year to distribute £80,000, including the £1,000 which the London Parochial Charities generously continue to entrust to us for allotment to convalescent homes. This sum is, I am only too well aware, insufficient to enable us to fulfil our original object. We could easily distribute £150,000 a year to hospitals which require the money and would put it to good use. In the meantime I venture to think that a means may be found of increasing the amount at the disposal of the hospitals for carrying on their ever-growing work.

### Decreased Expenditure Advocated.

It must be remembered that if the expenditure of the hospitals can be decreased without impairing the extent or efficiency of the work they do, the result would be even more satisfactory than an increase in the money subscribed to support the hospitals. (Hear, hear.) Waste of all kinds is a bad thing, and, as I have before pointed out, some of the hospitals certainly fail in the point of economy. This is a matter of the utmost importance, and one with which this Fund most properly concerns itself. Our object, however, is to help, not to hinder—to encourage, not to chill—the work of charity, and to do this by offering friendly assistance to those hospitals which, as we think, might save money by a few slight alterations in their arrangements. I feel sure those who are generously giving their time to the administrative work of our hospitals would accept the advice we offer in the spirit in which it is meant, even though on examination it might not be found to apply to their particular case. This year a beginning has been made in the direction I have indicated. A statistical report has been prepared on the expenditure of the 16 largest general hospitals which alone received more than half our grants last year, and in doing so we have supplied ourselves with information which no one hospital could be expected to provide on its own account. That report shows some remarkable results. It gives the average expenditure of these 16 hospitals on those branches of expenditure over which control exists, and the aggregate value of the sums paid in excess of these averages works out to no less than £39,400 for the year 1903. We do not say that that sum can all be saved at once. But no doubt every hospital will realise that a large saving could be effected by making sure that no other institution is, in similar circumstances, doing the same work as its own equally well and at a lower cost. (Hear, hear.)

### How to Reduce Prices.

We have done all in our power to provide the necessary information by giving the comparative prices of the principal goods used in the different hospitals. Some of the discrepancies are certainly worth serious consideration. By way of example I would mention that there is a difference of no less than 3¼d. per lb. between the highest and lowest prices paid for the same description of beef, of 3½d. per lb. for legs of mutton, of 1s. 1d. in the price of fowls, of 2½d. per gallon in the price of milk, of 7d. per lb. in the price of tea. In drugs and household articles there are similar discrepancies. There may be reasons to account for some of these differences, and all we ask for is careful inquiry on the part of each hospital. I am glad to hear that in the case of one of the largest and best managed hospitals our efforts have been gratefully acknowledged, and that, as a result of the comparisons we have provided, various economies have been effected which have resulted in a considerable saving. (Hear, hear.) With regard to the question of prices paid it is obvious that a large contract has a considerable advantage over a small one. For this reason I merely venture to throw out, as a suggestion, the possibility of some of the smaller hospitals combining to make a large contract, and thus gaining the advantage enjoyed by the larger institutions. If this is feasible, and the assistance of some outside body is required to organise the preliminary arrangements, I am quite certain that our Committee would gladly give every assistance in their power by encouraging hospital managers to meet and discuss the question.

### The Hospitals and the Medical Schools.

There is another matter to which I must briefly allude. The anonymous donor, in his letter to me, referred to the question of money subscribed to the relief of the sick poor being used for the purposes of medical education, and he expressed a hope that as President, I would endeavour to prevent any of the Fund being thus diverted. The doubts which evidently exist in the mind of the anonymous donor, were also shared by some other members of the Fund. The question is one which cannot be settled off-hand. For there are proper considerations which may be urged, both for and against a contribution from the general funds of a hospital, to the funds of its medical school. We have, therefore, thought it best to appoint a Committee, consisting of Lord Welby, Sir Edward Fry, and the Bishop of Stepney. I am glad to say that Committee has commenced its labours; that it is receiving every assistance from those who are able to set all sides of the question before it, and I feel confident that a report by three such eminent men will be of the greatest value in assisting us to come to a right conclusion.

### Guarantees Afforded by the Fund.

We have therefore shown our subscribers that we are endeavouring, as far as possible, to assist the hospitals to lay out our grants economically, and to husband their resources, that we are willing to help them, if they so desire, to settle any vexed question that may arise with regard to difficulties of administration whether internal or external, or on matters concerning reconstruction, removal, or amalgamation. The question remains, can we increase our income and therefore their income? I must own myself greatly disappointed at the results of our appeal for the sum necessary to enable us to accept the generous offer of my anonymous friend. Whether he may be willing to extend, beyond the end of this year, the period in which we may collect for this object, I cannot say. I trust he may do so, for I confess that there is to my mind no better form of charity; and I would, while we are still in 1904, appeal once more to those who desire to see their gifts economically and wisely distributed to help us to accept the offer. In these times of depression in trade there are, I know well, great difficulties. But I am convinced that sooner or later the working of this Fund will be appreciated at the proper value. I trust sooner.

### The President's Thanks.

As your President I wish to thank all the members of the Visiting Committee for their most valuable reports. Also the members of the Distribution Committees. I know their labours have been complex and often onerous. But I trust none of them will grudge the days given up from their business to the service of the Fund. The work of compiling the Statistical Report has been very severe. I must take this opportunity of conveying to the honorary secretaries, and especially to Mr. Danvers Power, what I am sure all privileged to see the report will desire, to express our warmest thanks and appreciation of the statistics so accurately compiled. (Hear, hear.)

### H.M. The King's Message.

The King wishes me to assure you of his unabated interest in the work of the Fund which he founded. His Majesty is gratified at the considerable advance made by the League of Mercy, and has noted with satisfaction that in the year's contribution one of the largest increases has been in a district, the Lady President of which is a near member of his family.

I beg now to move the adoption of the report. (Hear, hear.)

The Lord Mayor of London (Mr. John Pound) had pleasure in seconding the adoption of the report, which had been so ably moved by his Royal Highness the Prince of Wales.

Mr. Edgar Speyer supported the adoption of the report and said they were all grateful for the manner in which the work had been done. As far as the saving of money was concerned, he was sure that they could not too strongly express themselves in regard to hospitals being economically and efficiently managed. It was essential that the business methods adapted to the work of the world should be applied to hospital management also.

### St. Bartholomew's Hospital.

Sir Joseph Dimsdale said:—He understood from the report read by Sir William Church that it did not come within the province of the Fund to give to any hospital which is substantially endowed. On that ground St. Bartholomew's Hospital was entirely eliminated, but he found many hospitals with endowments were assisted. He ventured to think he might be permitted to ask his Royal Highness where the inconsistency was in not recognising the claims of a hospital which for 150 years had not appealed to the public for a single farthing and was now only appealing to put the fabric of the building into a state that would bring it up to modern requirements.

Mr. H. C. Smith, as chairman of the Executive Committee, said:—In answer to Sir Joseph Dimsdale, he would like to say that the matter was referred by the Distribution Committee to the Executive Committee, and they gave patient consideration to it. They thought that a hospital possessed of such very large property as St. Bartholomew's was not one coming within the scope of the Fund. He believed that St. Bartholomew's possessed property valued at over £2,000,000 sterling. They knew that for many years no appeal for funds had been made to the public, and they conceived that if St. Bartholomew's was again fully endowed it would not require assistance from the Fund or the public. Therefore, supposing that a substantial grant were made at the present time, it would not carry with it the right of future inspection and recommendation—the indispensable condition upon which the Fund's moneys are distributed.

The resolution was then put and carried unanimously.

It was decided that the cheques should be posted before December 22nd.

### "An Enormous Amount of Thought and Work."

The Bishop of Rochester said he begged to propose a vote of thanks to his Royal Highness for presiding. He had his own personal reasons for thanking his Royal Highness in this matter, because he knew what the Committee had been able to do to assist in transferring King's College Hospital to South London, and to those in South London this step was a matter of profound gratification. Speaking, however, as a lay member of the Council, and having nothing to do with the work of its Committees, he thought it was impossible for anyone to listen to what had been said and read of the reports of the various Committees without seeing what an enormous amount of thought and searching work was now being done by these Committees over which his Royal Highness presided. (Hear, hear.) It would seem to him that through the creation of this Fund by his Majesty the King, and its inheritance by the Prince of Wales, the effect had been that public charity had obtained the power to inquire into the conduct of the hospital work of the metropolis, which must be of the highest public benefit. This right, as it were, of inquiry which was certainly exercised in the most kindly and sympathetic way, but which must secure results of efficiency, economy, and centralisation up to a point, must attract public attention. He believed we should see that in addition to the spring of charity within the human breast there was nothing so powerful for collecting funds as to be able to give an assurance of economy and efficiency on the part of those agencies which the public were asked to assist. He did not think anyone could have sat at that board to-day and listened to what his Royal Highness had laid before them, without realising that this is the result which the King's Hospital Fund is now in a greatly increasing measure insuring for the public. (Hear, hear.)

Lord Iveagh seconded the vote of thanks to his Royal Highness, the vote being unanimously carried.

His Royal Highness said: My Lords and Gentlemen, I beg to thank you for the kind vote of thanks, and to say again that I shall always take the greatest possible interest in this Fund. When I see so many friends around me, I wish again to assure them how deeply I appreciate their valuable services.

The proceedings then terminated.

